# Utilization of the Cervical Flexion Rotation Test to Confirm Rotation Directional Preference in People With Neck Pain: A Case Series

**DOI:** 10.7759/cureus.47389

**Published:** 2023-10-20

**Authors:** Michael D Post, Ronald J Schenk, Ross Fargnoli

**Affiliations:** 1 Physical Therapy, Good Shepherd Penn Partners, Philadelphia, USA; 2 Physical Therapy, Tufts University School of Medicine, Boston, USA

**Keywords:** neck pain, upper cervical spine, directional preference, mckenzie method, mechanical diagnosis and therapy, cervical flexion rotation test

## Abstract

Despite the prevalence of neck pain, evidence is lacking regarding the relationship of pathophysiology to function in people with neck conditions. Although movement-based diagnoses based on directional preference (DP) are described for lumbar spinal conditions, how these diagnoses guide interventions is not supported in the Cervical Spine Clinical Practice Guidelines. To date, there are no case studies in the literature that demonstrate the efficacy of cervical spine management based on a rotation DP. This case series highlights patient response to repeated end-range neck movements to inform DP and how the cervical flexion rotation test (CFRT) was used as a clinical baseline to assess mechanical and symptomatic changes. Three consecutive patients were evaluated by a physical therapist fellow trained in orthopedic manual physical therapy and diplomaed in mechanical diagnosis and therapy. The patients’ baseline pain ranged from 3 to 7/10 on the Numerical Pain Rating Scale (NPRS), and disability scores ranged from 20% to 52.6% on patient-reported outcome (PRO) measures. All three cases demonstrated a limited and painful CFRT. Examination procedures included repeated end-range movement testing in the sagittal and frontal and transverse planes. Across five to six visits in five to eight weeks, a decrease in the primary outcome measures from baseline to discharge were observed: NPRS, 50-85%; PRO, 60-82%. The CFRT may be a key baseline when screening patients with neck pain for DP. Following repeated end-range sagittal and frontal plane movements, the rapid change in the CFRT following targeted upper cervical rotation techniques confirmed a rotation DP.

## Introduction

Neck pain is among the most burdening, costly, and prevalent musculoskeletal conditions [1,2]. The point prevalence of neck pain has been reported approximately 5-39% with a lifetime prevalence of 1-71% [2,3]. The incidence of cervical radiculopathy is less common, with a prevalence of approximately 1-7% [4]. Pre-validation studies have attempted to coordinate diagnostic testing with pain mechanism-based classifications, but treatment decisions based on the pathoanatomical source of neck pain are not fully supported in the literature [5]. Evidence is lacking regarding the identification, prevalence, validity, or relationship of pathophysiology to pain and function in those with neck pain [6].

Directional preference (DP) is a movement-based diagnosis subtype and a phenomenon researched for those with spinal pain [7]. DP is defined as a specific direction of movement that positively affects movement and either decreases, centralizes, or abolishes pain symptoms. A DP movement may be those opposite to the movement, which results in an increase in pain or restricted range of movement. Given the lack of evidence correlating pathophysiology and function, movement-based diagnoses are becoming increasingly popular for guiding rehabilitation for neck conditions [8]. Although movement-based diagnoses based on DP are described for lumbar spinal conditions, DP is not referenced in the Cervical Spine Clinical Practice Guidelines [8,9].

The McKenzie Method of Mechanical Diagnosis and Therapy (MDT) is a reliable and valid system to assign a movement-based diagnosis and treat neck pain based on the clinical response to patient- and clinician-generated procedures [7,10]. This method involves the assessment of symptomatic and mechanical baselines before, during, and after repeated end-range movement testing to classify patients into one of four syndromes: derangement, dysfunction, postural, and others [7]. Repeated end-range movement testing, typically initiated with sagittal plane movements and exploring frontal and transverse plane movements as needed, may determine DP. Centralization, a subtype of DP, is confirmed when spinal-referred symptoms abolish in a distal to proximal pattern in response to repeated end-range movements or sustained postures [7]. DP and centralization are characteristic of the derangement syndrome classification and have been shown to lead to favorable outcomes in those with heterogeneous neck pain [10]. The prevalence of these findings in those with neck pain has been reported in the literature: derangement syndrome classification, 92%; centralization, 74-82%; DP to extension, 78.7%; and DP to lateral movements, 13.7% [11]. Although neck rotation DP exercise is utilized clinically, there are presently no case-series reports in the literature demonstrating the efficacy of cervical spine management based on a rotation DP alone.

The cervical flexion rotation test (CFRT) is a useful clinical test for determining movement limitations in the upper cervical spine [8]. This passive movement test of end-range cervical flexion followed by end-range upper cervical rotation may be a key clinical baseline to identify the cervical derangement syndrome with a relevant lateral rotation DP. The purpose of this case series is to describe the outcomes of three patients with neck pain who were managed with cervical rotation as their DP.

## Case presentation

Methods

Three consecutive de-identified patients classified as a cervical derangement, according to the MDT principles, who presented with a positive CFRT were treated with upper cervical rotation mobilization and muscle energy techniques. The manual procedures were followed with cervical rotation exercises. A clinician with a doctorate in physical therapy, diploma level in MDT, and fellow of the American Academy of Orthopedic Manual Physical Therapists performed the examination and interventions procedures with the three patients.

These examinations consisted of the following tests and measures: a review of constitutional, cardiovascular, integumentary, musculoskeletal, and neurological systems; past medical history; imaging; and assessment of body structures and functions, including screening for contraindications and barriers to exercise or manual therapy procedures. The examination procedures determined normal findings for upper-extremity deep tendon reflexes, Hoffman’s reflex, C3-T1 myotomes and dermatomes, and distraction, compression, and Spurling’s tests. The physical examinations also included testing repeated end-range movements of the cervical and thoracic spine with the monitoring of symptomatic and mechanical responses. Provided that all three demonstrated DP, direction-specific patient-generated repeated end-range movements were prescribed as home exercises. Follow-up examinations included assessment of range of motion and patients' self-report of pain and function.

**Figure 1:**
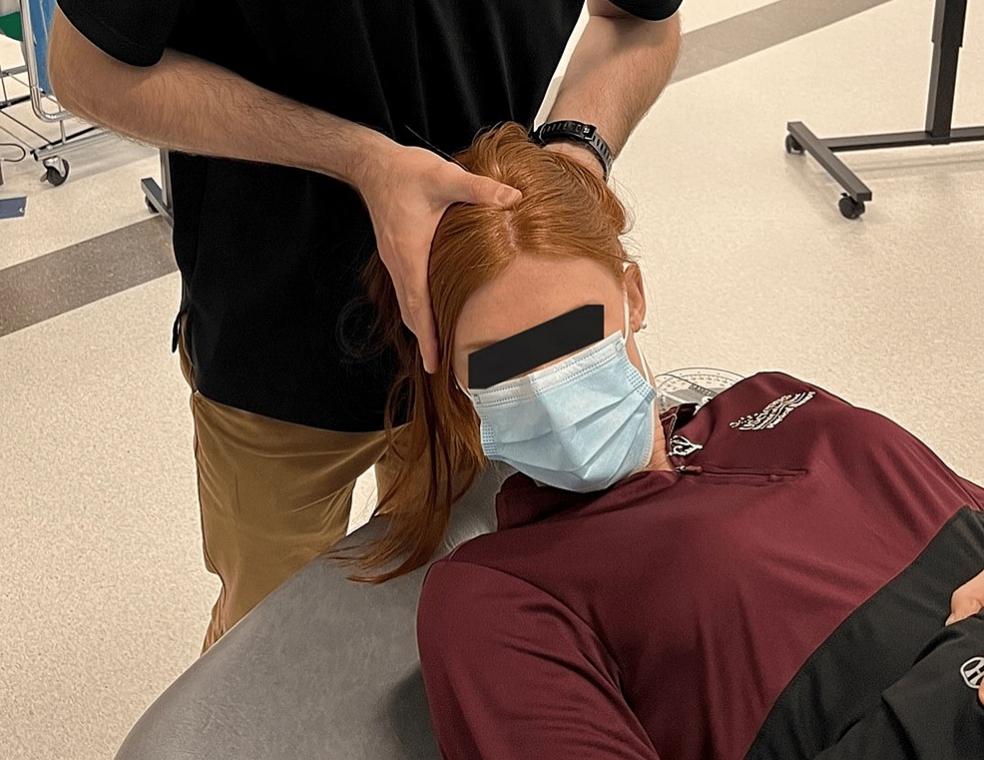
Cervical flexion rotation test

**Figure 2:**
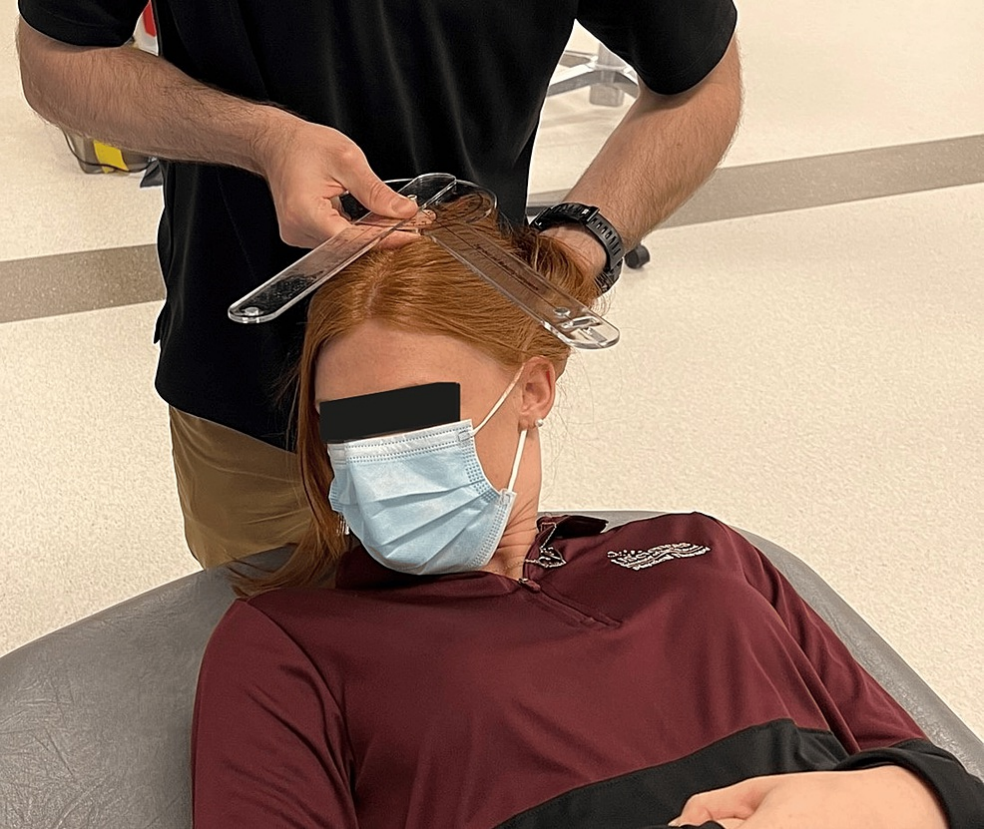
Cervical flexion rotation test with goniometry

Range of motion was recorded based on the patient's active and passive procedures by clinician interpretation of goniometry, inclinometry, or by nil/min/mod/maj loss defined within the MDT examination nomenclature [7,8]. Although visual observation is a reliable method of examination of the CFRT, a goniometer was utilized for case 3 (Figure 1, Figure 2) [8,12]. Upper limb tension test 1 (ULTT1) was recorded as degrees of elbow extension.

The patient self-report measures included the following: 1) Numerical Pain Rating Scale (NPRS); 2) Global Perceived Effect (GPE) or Global Rating of Change (GROC) scale; and 3) Neck Disability Index (NDI) or the Quick-Disabilities of the Arm, Shoulder, and Hand (QuickDASH). These are valid measures with established minimum detectable change (MDC) and minimum clinically important differences (MCID) [13-17]. Statistically significant change in these has been reported as a change of +/-2/10 on NPRS, +/-5 points on GROC, 19% on NDI, and 15% on QuickDASH [13-15,17,18].

This report was exempt from Institutional Review Board appraisal and was approved by the University of Pennsylvania Institutional Review Board, Philadelphia, USA.

History and function

Case 1

A 25-year-old male referred to physical therapy for cervical radiculopathy presented with a one-year history of insidious, intermittent, bilateral, asymmetrical left-sided neck pain and pain in the left scapular, shoulder, and upper arm regions. The NPRS was rated as 3/10 at worst and described as aching, dull, and tight. Symptoms worsened with neck movement and prolonged static activities and improved with recumbent positioning and heat. Radiographs were unremarkable. The patient reported good general health with no significant past medical history.

Case 2

A 38-year-old male referred to physical therapy for cervical radiculopathy presented with a five-week duration of asymmetrical, unilateral, left-sided neck, scapular, shoulder, and upper arm pain, which onset after repetitive lifting activities. NPRS was 4/10 at worst and described as aching, dull, and tight. Symptoms worsened with driving. He was previously examined by another physical therapist and was instructed to perform repeated cervical left lateral flexion with self-overpressure as a home program, with which the patient reported short-term pain relief. He denied other positions or activity, which improved symptoms. No diagnostic imaging was completed prior to examination. The patient reported good general health with no significant past medical history.

Case 3

A 76-year-old female referred to physical therapy for cervicalgia presented with a one-year duration of insidious, unilateral, left-sided neck pain. NPRS was 7/10 and described as aching and tight. Symptoms worsened with left neck rotation. She did not report positions or activities that improved her symptoms. Self-reported quality of life was excellent, and her past medical history included sleep apnea, coronary artery disease, gastroesophageal reflux, mild persistent asthma, peripheral neuropathy, restless legs syndrome, prediabetes, and urinary incontinence. At the time of examination, she was also receiving physical therapy services for balance deficits. No diagnostic imaging was completed prior to examination. 

Examination and observation findings

A summary of examination findings for each individual case are presented on Table 1, Table 2, and Table 3. Seated active movement assessment was completed at the cervical spine for all three cases, with a significant asymmetrical cervical rotation toward the affected side in Cases 2 and 3. Special testing revealed a positive CFRT and limited and painful deep neck flexor endurance testing in all three cases. Additional significant findings included ULTT1 in Cases 1 and 2.

Intervention, follow-up, and outcomes

Case 1

Initial assessment explored repeated cervical movements in the sagittal plane, including retraction and extension movements with overpressure and manual therapy procedures (Table 1). Response to repeated end-range movements led to assessment of cervical retraction with extension and clinician overpressure, resulting in improved cervical ROM and ULTT1. Based upon the positive observed changes to clinical baselines, further testing was stopped. The patient’s home exercise program (HEP) included cervical retraction with extension and self-overpressure to be completed throughout the day. The patient was advised to discontinue the exercise if he experienced peripheralization of pain or worsening symptoms.

**Figure 3:**
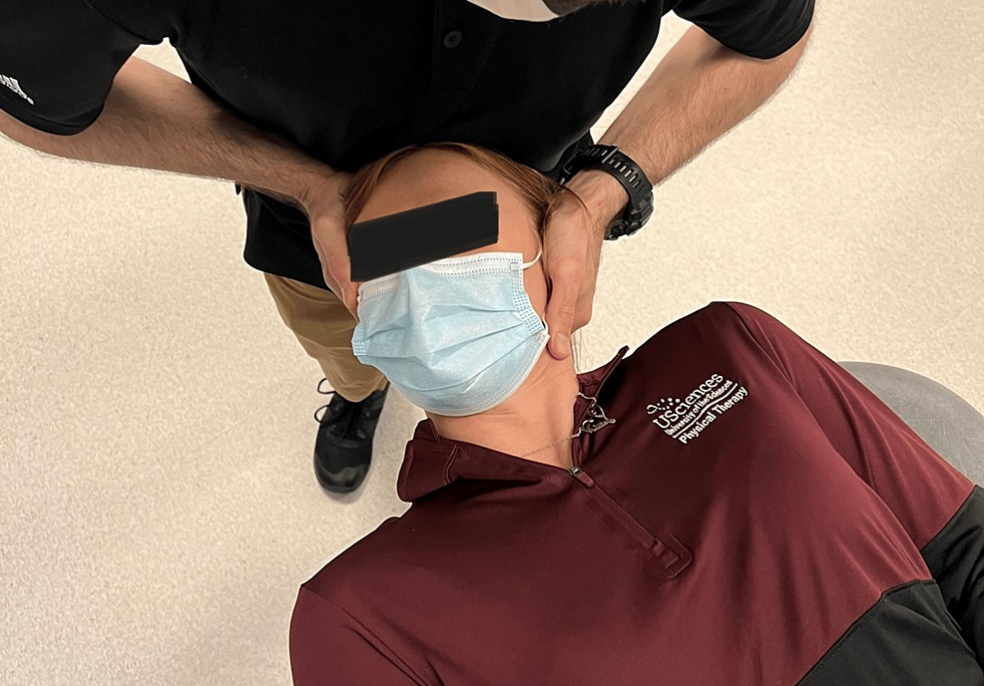
Supine right upper cervical rotation clinician mobilization Position is the same for thrust mobilization or muscle energy technique.

**Figure 4:**
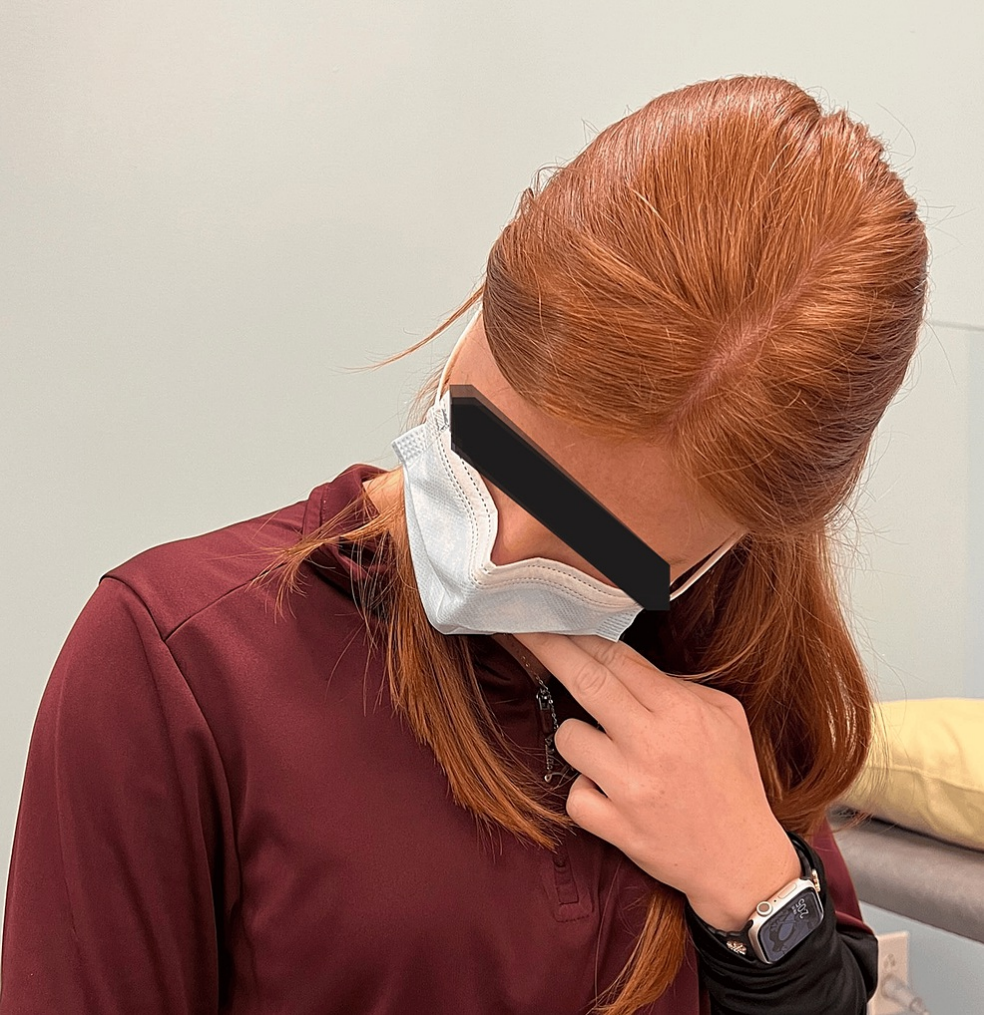
Right upper cervical rotation in flexion with self-overpressure

The patient returned seven days later for visit 2. He reported improvements in GPE and pain, demonstrated improved ULTT1 symmetry, and improved left cervical rotation by 10 degrees (Table 1). Further assessment of repeated cervical sagittal and frontal plane movements, following the MDT system algorithm, resulted in no additional improvements, so transverse plane procedures were explored. Following right upper cervical rotation clinician mobilizations (Figure 3), the patient showed improved right cervical rotation ROM and CFRT, indicating that he responded positively to this intervention. The patient’s HEP was modified to repeated right cervical rotation in flexion with self-overpressure (Figure 4). 

**Table 1:** Interventions and patient response (Case 1) ROM: range of motion; ULTT1: upper limb tension test 1; PRO: patient-reported outcome; NPRS: Numerical Pain Rating Scale; FLEX: flexion; EXT: extension; LLF: left lateral flexion; RLF: right lateral flexion; LROT: left rotation; RROT: right rotation; CFRT: cervical flexion-rotation test; ULTT1: upper limb tension test 1; R: right; L: left; GPE: global perceived effect; MET: muscle energy technique

Visit (week)	Between-session response	Intervention (sets x reps)	Within-session response	Home exercise program
1 (week 1): Initial examination	Baseline pain and function: 3/10; self-reported function 60%; NDI 34%	Seated cervical retraction with clinician overpressure, 3x5	Produced neck pain, not worse; increased cervical ROM; plateaued	Seated cervical retraction with extension and self-overpressure, x10-20 reps, 4-6 times/day
	Baseline ROM: FLEX, min loss; EXT, min loss; LLF, nil loss; RLF, nil loss; LROT, 70°; RROT, 70°	Seated cervical retraction with clinician mobilization, 2x3	Produced neck pain, not worse; cervical ROM increased; plateaued	Education: peripheralization of pain or muscular weakness
	Baseline special tests: positive: CFRT (R); ULTT1 L 152 deg; ULTT1 R 165 deg; DNF endurance test 3 sec	Seated cervical retraction with extension and clinician overpressure, 3x5	Produced neck pain, not worse; ULTT1 ROM increased	
2 (week 2)	Symptoms: NPRS 3/10	Seated cervical retraction with clinician overpressure, 3x5	Increased neck pain, not worse; no effect to ROM	Upright right upper cervical rotation in cervical flexion with self-overpressure, x10-20 reps, 4-6 times/day
	Key baselines: EXT/LLF/RLF, nil loss; FLEX, min loss; LROT, 80 deg; RROT, 70 deg; ULTT1 symmetrical ROM, tight L	Supine cervical retraction and extension with clinician traction and overpressure, 2x3	Produced neck pain, not worse; no effect to ROM	Education: peripheralization of pain or muscular weakness
	PRO: GPE 15% improved; self-reported function 80%	Supine cervical flexion with clinician mobilization, 2x3	Produced neck pain, not worse; ULTT1 tightness improved; plateaued	
		Seated cervical right lateral flexion with clinician mobilization, 2x3	No effect on pain; no effect ROM	
		Supine right upper cervical rotation with clinician mobilization, 2x3	Produced neck pain, not worse; right cervical rotation increased to 80 deg, CFRT improved symmetry	
3 (week 3)	Symptoms: NPRS 1/10	Supine right upper cervical rotation with clinician mobilization, 4x3	Produced tightness, not worse	Upright right upper cervical rotation in cervical flexion with self-overpressure, x10-20 reps, 4-6x/day
	Key baselines: FLEX/EXT/LLF/RLF, nil loss; LROT, 85 deg; RROT, 85 deg; ULTT1 symmetrical	Supine right upper cervical rotation antagonist MET, 7 sec, 3x7	Produced tightness, not worse	Education: discontinue if symptoms worsen
	PRO: GPE 50% improved; self-reported function 85%			
4-5 (weeks 4-6)	Symptoms: NPRS 1/10	Supine right upper cervical rotation with clinician mobilization, 4x3	Produced tightness, not worse	Upright right upper cervical rotation in cervical flexion with self-overpressure, x10-20 reps, 4-6x/day
	Key baselines: FLEX/EXT/LLF/RLF, nil loss; LROT, 85 deg; RROT, 85 deg; ULTT1 symmetrical; DNF endurance 38 sec	Supine right upper cervical rotation antagonist MET, 7 sec, 3x7	Produced tightness, not worse	Scapulothoracic therapeutic exercise: prone lying with shoulder press, bird dog, resisted shoulder flexion and diagonal-pattern flexion; 3 sets x20 reps each
	PRO: GPE 95% improved; self-reported function 95%; NDI 6%	Scapulothoracic therapeutic exercise: prone lying with shoulder press, bird dog, resisted shoulder flexion and diagonal-pattern flexion; 3x20 each		Education: continue exercises next 2-4 weeks, or as needed

**Figure 5:**
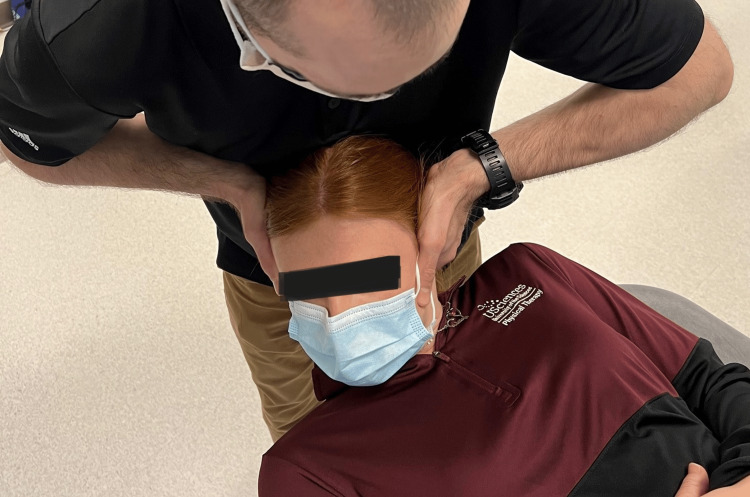
Supine right upper cervical rotation clinician mobilization from flexion preposition Position is the same for thrust mobilization or muscle energy technique.

The patient returned seven days later for visit 3. He reported improvements in GPE and pain and demonstrated within-normal symmetrical cervical ROM and ULTT1. Pain persisted at repeated end-range right cervical rotation. Based upon the observed improvements in clinical baselines, right upper cervical rotation clinician mobilizations were continued, and antagonist muscle energy technique procedures were performed to facilitate improved right cervical rotation (Figure 5). The patient was instructed to continue his previous HEP in order to maintain improvements.

**Figure 6:**
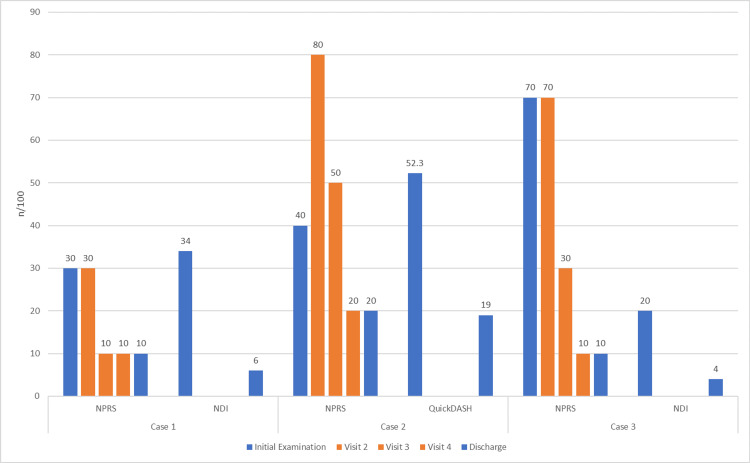
Pain and patient-reported disability outcomes from initial examination to discharge n: number; NPRS: Numerical Pain Rating Scale; NDI, Neck Disability Index; QuickDASH, Quick Disabilities of the Arm, Shoulder, and Hand

The patient returned for visits 4 and 5 at two-week intervals. Through visit 5, he reported a GPE of 95% improvement and self-reported function of 95%, and the NPRS was rated as 1/10 at worst. Cervical ROM was without loss and ULTT1 remained symmetrical and without significant tightness. Deep cervical flexion endurance testing improved from three seconds at evaluation to 38 seconds before fatigue and without pain. Prior to discharge, the patient was guided to perform scapulothoracic movement control and strengthening exercises while monitoring maintenance of improvement and recovery of functional activity tolerance. At discharge, the patient met the MCID for all outcome measure scores and was independent with his HEP (Figure 6, Table 1).

Case 2

Initial assessment included repeated end-range cervical movements in the frontal plane to assess his response to the previously prescribed HEP (Table 2). Reassessment of patient response indicated improved upper arm pain and cervical ROM; however, improvements plateaued. Due to the lack of improvement, the target examination treatment plane was altered at this time to assess the patient’s response to sagittal plane movements. Repeated slouch-overcorrect exercise was performed to assess symptoms and assess his ability to maintain an upright posture, which resulted in reduced upper arm pain, improved cervical ROM, and improved ULTT1. Based upon changes in clinical baselines, further testing was stopped. The patient’s HEP was updated to include cervical left lateral flexion with self-overpressure and slouch-overcorrect posture during sitting throughout the day. The patient was advised to discontinue the exercise if he experienced peripheralization of pain or worsening symptoms.

The patient returned four days later for visit 2. He reported a gradual worsening of pain and GPE. Despite these subjective reports, his cervical ROM and ULLT1 were improved. Review of his HEP and re-assessment of repeated frontal plane movements did not result in any additional improvements. Based on this lack of change, the effect of repeated end-range sagittal plane extension procedures was assessed, which resulted in no additional benefit. Cervical flexion procedures resulted in abolished upper arm pain, improved cervical ROM, and improved movement associated pain. Based upon the positive changes to clinical baselines and symptoms, further testing was stopped. The patient’s HEP was modified to repeated cervical flexion with self-overpressure to be completed throughout the day.

The patient returned seven days later for visit 3. He reported improvements in GPE and pain and demonstrated improved and symmetrical ULTT1. He continued to report asymmetrical and painful left cervical rotation. Reassessment of cervical flexion procedures did not result in any additional improvement. Since he continued to have limited cervical rotation ROM and a positive left CFRT, left cervical rotation clinician mobilizations targeting the upper cervical spine were performed; this resulted in improved CFRT. Following procedures, antagonist muscle energy techniques were performed to facilitate improved left cervical rotation. Based upon positive changes in ROM and movement associated pain, additional testing was stopped. The patient’s HEP was modified to include repeated left cervical rotation in flexion or contralateral flexion, with self-overpressure (Figure 2C, 2D).

**Figure 7:**
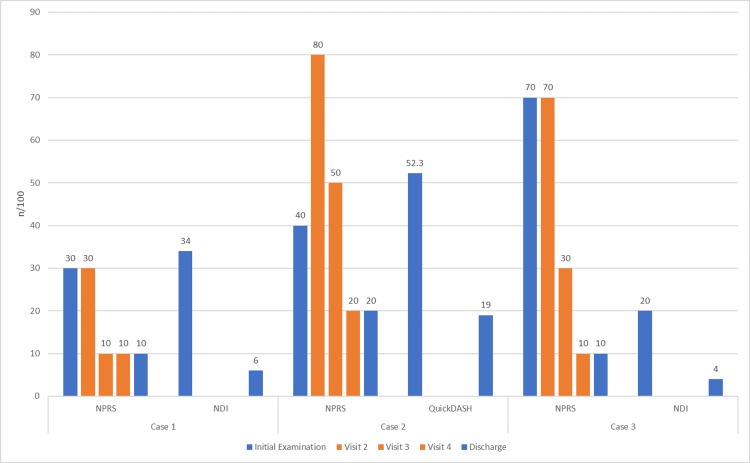
Pain and patient-reported disability outcomes from initial examination to discharge n: number; NPRS: Numerical Pain Rating Scale; NDI: Neck Disability Index; QuickDASH: Quick Disabilities of the Arm, Shoulder, and Hand

The patient returned for visits 4 and 5 at one and two-week intervals. Through visit 5, he reported a GPE of 80% improvement and self-reported function of 90%, and NPRS was 2/10 at worst. Cervical ROM and ULTT1 were normal and symmetrical. Prior to discharge, the patient was guided to begin performing deep neck flexor endurance training and upper body strengthening while monitoring maintenance of improvement and recovery of functional activity tolerance. At discharge, the patient met the MCID for all outcome measure scores and was independent with his HEP (Figure 7, Table 2).

**Table 2:** Interventions and patient response (Case 2) ROM: range of motion; ULTT1: upper limb tension test 1; PRO: patient-reported outcome; NPRS: Numerical Pain Rating Scale; FLEX: flexion; EXT: extension; LLF: left lateral flexion; RLF: right lateral flexion; LROT: left rotation; RROT: right rotation; CFRT: cervical flexion-rotation test; ULTT1: upper limb tension test 1; R: right; L: left; GPE: global perceived effect; MET: muscle energy technique

Visit (week)	Between-session response	Intervention (sets x reps)	Within-session response	Home exercise program
1 (week 1): Initial Examination	Baseline pain and function: 4/10; self-reported function 60%; QuickDASH 52.3%	Seated cervical left lateral flexion with self-overpressure, 2x10	Decreased upper arm pain, better; cervical ROM increased	Seated cervical left lateral flexion with self-OP and slouch-overcorrect posture in sitting, x10-20 reps, 4-6x/day
	Baseline ROM: FLEX, min loss; EXT, nil loss; LLF, min loss; RLF, min loss; LROT, 65°; RROT, 75°	Seated cervical left lateral flexion with clinician mobilization, 3x3	Decreased upper arm and neck pain, better; no effect ROM	Education: peripheralization of pain or muscular weakness
	Baseline special tests: positive: CFRT (L); ULTT1 L 144 deg; ULTT1 R 157 deg; DNF endurance test 8 sec	Seated slouch-overcorrect, 2x10	Decreased upper arm and neck pain, better; ULTT1 ROM increased	
2 (week 1)	Symptoms: NPRS 8/10	Seated cervical left lateral flexion with self-overpressure and seated slouch-overcorrect 1x10 each	No effect on pain; no effect ROM	Seated cervical flexion with self-OP, x10-20 reps, 4-6x/day
	Key baselines: RLF/LLF/PRO, nil loss; RET/EXT/FLEX, min loss; LROT, 80 deg; RROT, 90 deg; ULTT1 L, 155 deg; ULTT1 R, 160 deg	Seated cervical left lateral flexion clinician-mobilization, 3x5	No effect on pain; no effect ROM	Education: peripheralization of pain or muscular weakness
	PRO: GPE 50% worse; self-reported function 60%	Seated cervical retraction with extension and self-overpressure, 2x10	Increased upper arm pain, not worse; no effect ROM	
		Seated sustained cervical extension x30 sec	Increased upper arm pain, not worse; no effect ROM	
		Seated cervical flexion with self-overpressure, 2x10	Decreased upper arm pain; reduced movement associated pain with neck ROM, better	
		Supine cervical flexion clinician mobilization, 3x5	Abolished upper arm pain, better; cervical ROM increased	
3 (week 2)	Symptoms: NPRS 5/10	Seated cervical flexion with self-overpressure 1x10	No effect on pain; no effect ROM	Seated left upper cervical rotation with self-overpressure from a cervical flexed or contralaterally flexed position, x10-20 reps, 4-6x/day
	Key baselines: RET/EXT/LLF/RLF/PRO, nil loss; FLEX, min loss; LROT, 80 deg; RROT, 90 deg; ULTT1 L/R, 160 deg; CFRT L (+)	Supine cervical flexion clinician mobilization 3x5	No effect on pain; no effect ROM	Education: discontinue if symptoms worsen
	PRO: GPE 50% improved; self-reported function 60%	Supine left upper cervical rotation clinician mobilization, 5x5	CFRT L ROM increased	
		Supine left upper cervical rotation MET with reciprocal inhibition, 3-5 sec, 3x7	Produced tightness, not worse	
4-5 (weeks 3-4)	Symptoms: NPRS 2/10	Seated left upper cervical rotation from a cervical flexed position, x20	Produced tightness, not worse	Seated left upper cervical rotation with self-overpressure from a cervical flexed or contralaterally flexed position, x10-20 reps, 4-6x/day
	Key baselines: all cervical ROM, nil loss; ULTT1 L/R, symmetrical; DNF endurance 8 sec	Supine left upper cervical rotation clinician mobilization, 5x5	Produced tightness, not worse	Therapeutic exercise: standing shoulder flexion with resistance bands, modified push-up, DNF endurance training, 3x20 each for 2-4 weeks or as desired
	PRO: GPE 80% improved; self-reported function 90%; QuickDASH 19%	Supine left upper cervical rotation MET with reciprocal inhibition, 3-5 sec, 3x7		
		Therapeutic exercise: standing shoulder flexion with resistance bands, modified push-up, DNF endurance training, 3x20 each		

Case 3

**Figure 8:**
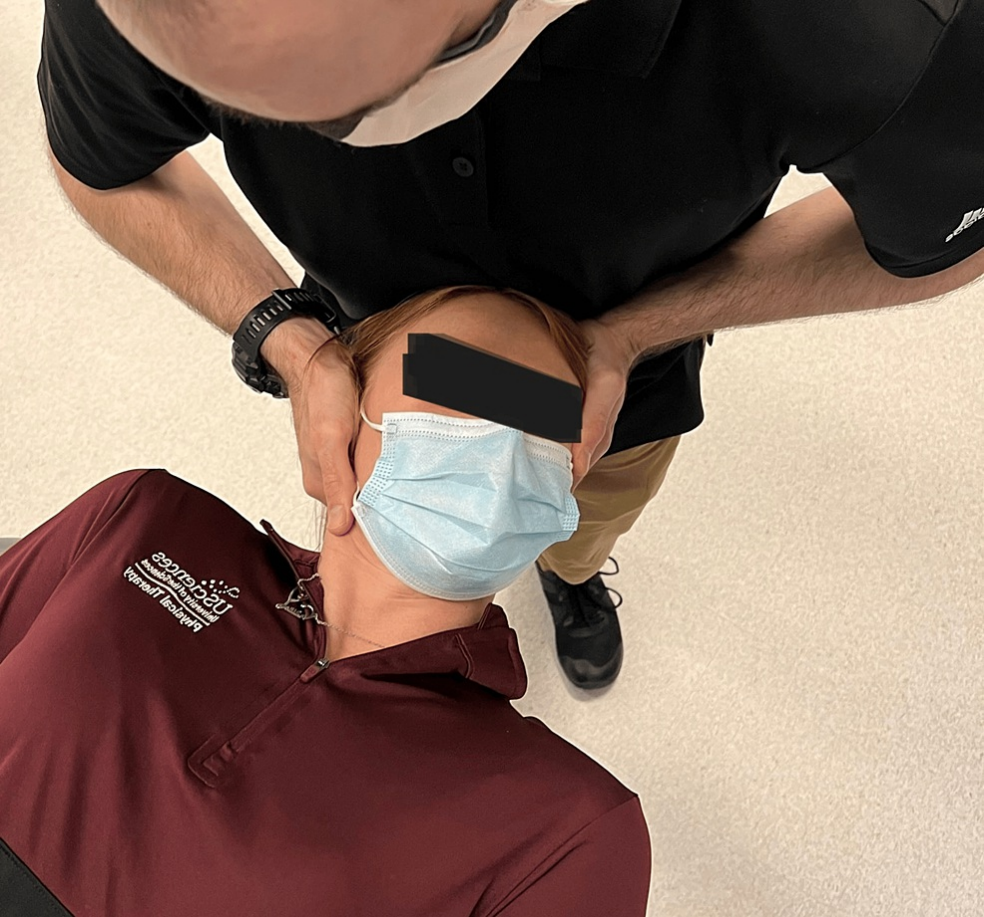
Supine left upper cervical rotation clinician mobilization Position is the same for thrust mobilization or muscle energy technique.

Initial assessment included repeated cervical movements in the sagittal and frontal planes. This included repeated end-range extension and flexion movements and lateral flexion movements, with clinician overpressure and mobilization procedures (Table 3). Following no significant changes to clinical baselines, transverse plane procedures were assessed. Left upper cervical rotation clinician mobilizations (Figure 8) were performed, resulting in improved cervical ROM. Based upon this positive change, further testing was stopped. The patient’s HEP included repeated cervical rotation in flexion or contralateral flexion, with self-overpressure. The patient was advised to discontinue the exercise if she experienced increasing or worsening pain.

**Figure 9:**
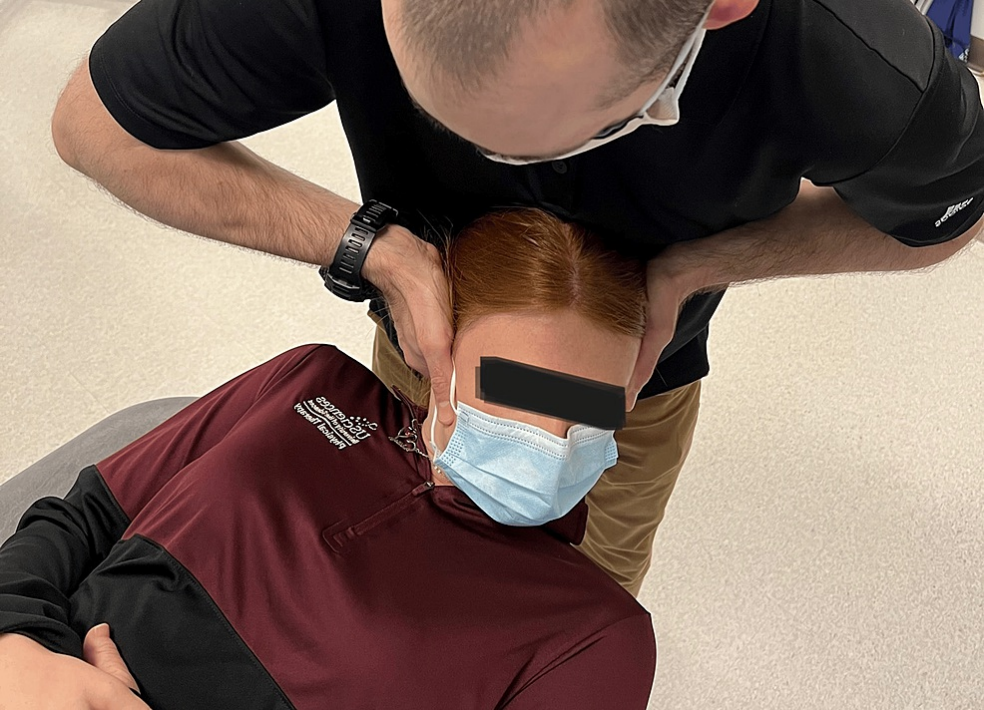
Supine left upper cervical rotation clinician mobilization Position is the same for thrust mobilization or muscle energy technique.

The patient returned 10 days later for visit 2. She reported 50% HEP adherence and 7/10 pain at worst. She demonstrated improved left cervical rotation ROM, but the left CFRT remained positive. Initially, reassessment of manual left upper cervical rotation clinician mobilization, prepositioned in right lateral flexion, resulted in increased neck pain and decreased cervical rotation ROM. The mobilization was then modified and performed in a cervical flexion preposition (Figure 9), resulting in improved ROM back to baseline. After reaching a plateau with increased repetitions, progression of forces was considered. The patient consented to receive a thrust mobilization prepositioned in flexion targeting the upper cervical spine (Figure 9), resulting in improved cervical ROM and movement associated pain. Antagonist muscle energy technique procedures were then performed to facilitate improved left cervical rotation. Based upon within-session improvements in ROM and movement associated pain, further testing was stopped. The patient’s HEP was modified to repeated cervical rotation movements in flexion, with self-overpressure, to be performed throughout the day. 

The patient returned for visits 3 and 4, at 6 and 21-day interval. She reported 75-90% HEP adherence, which alleviated her neck stiffness. NPRS was 3/10 at worst. She continued to demonstrate limited left cervical rotation ROM. However, her CFRT improved to symmetrical ROM, with left-sided stiffness. Based upon improvements in pain and mobility, manual therapy and muscle energy technique were continued and remained the same as the previous session. Supine deep cervical neck flexor endurance training was initiated utilizing a biofeedback cuff for visual feedback while monitoring patient maintenance of improvement and recovery of function. The patient’s HEP was modified to include supine deep cervical neck flexor endurance training into a pillow, 10 seconds hold for 10 repetitions, once per day.

**Figure 10:**
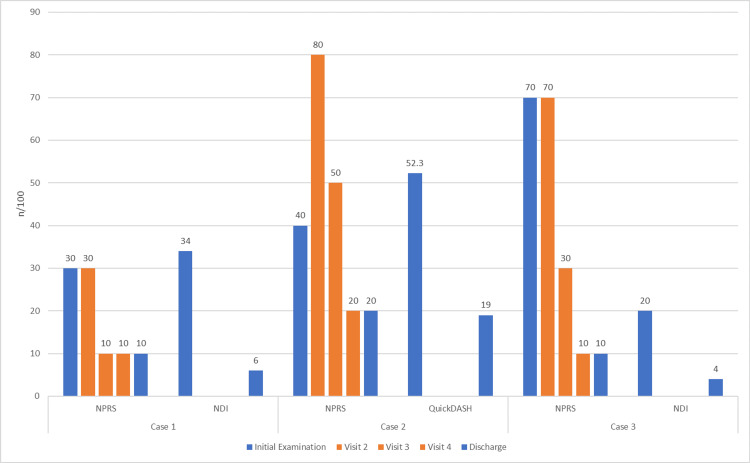
Pain and patient-reported disability outcomes from initial examination to discharge n: number; NPRS: Numerical Pain Rating Scale; NDI: Neck Disability Index; QuickDASH: Quick Disabilities of the Arm, Shoulder, and Hand

The patient returned for visits 5 and 6 at eight- and 11-day intervals. The NPRS improved to 1/10 at worst. Cervical rotation ROM remained limited to the left, but her CFRT mobility improved and remained symmetrical. Prior to discharge, the patient was guided to begin performing upper extremity and scapulothoracic strengthening exercises while monitoring patient maintenance of improvement and recovery of function. At discharge, the patient met the MCID for all of her outcome measure scores and was independent with her HEP (Figure 10, Table 3).

**Table 3:** Interventions and patient response (Case 3) ROM: range of motion; ULTT1: upper limb tension test 1; PRO: patient-reported outcome; NPRS: Numerical Pain Rating Scale; FLEX: flexion; EXT: extension; LLF: left lateral flexion; RLF: right lateral flexion; LROT: left rotation; RROT: right rotation; PRO: protraction; RET: retraction; CFRT: cervical flexion-rotation test; ULTT1: upper limb tension test 1; R: right; L: left; GROC: global rating of change; MET: muscle energy technique

Visit (week)	Between-session response	Intervention (sets x reps)	Within-session response	Home exercise program
1 (week 1): initial examination	Baseline pain and function: 7/10; NDI 20%	Seated cervical retraction with clinician overpressure, 3x5	Produced neck pain, not worse; no effect ROM	Seated left upper cervical rotation with self-overpressure from cervical flexion or contralaterally flexed position, x10-20 reps every 1-2 hours
	Baseline ROM: FLEX, 60°; EXT, 40°; LLF, 20°; RLF, 15°; LROT, 40°; RROT, 55°	Seated cervical retraction and extension with clinician overpressure, 3x5	Produced neck pain, not worse; no effect ROM	Education: discontinue if symptoms worsen
	Baseline special tests: positive: CFRT (L); DNF endurance test 0 sec	Prone posterior to anterior clinician mobilization to the low-mid-upper cervical spine, 2x5 each	No effect	
	Negative: ULTT1 L 153 deg; ULTT1 R 147 deg (limited by elbow ROM); VBI	Supine cervical flexion with clinician overpressure and mobilization, 4x5	Produced neck pain, not worse; no effect ROM	
		Seated cervical left lateral flexion with clinician overpressure and mobilization, 2x5 each	Produced neck pain, not worse; no effect ROM	
		Supine left upper cervical rotation clinician mobilization, 4x5	Produced neck pain, not worse; cervical ROM increased	
2 (week 2)	Symptoms: NPRS 7/10	Supine left upper cervical rotation clinician mobilization in right lateral flexion preposition, 4x5	Produced neck pain, worse; cervical rotation decreased	Seated left upper cervical rotation with self-overpressure in flexion, x10-20 reps every 1-2 hours
	Key baselines: EXT, 40 deg; FLEX, 70 deg; LLF, 25 deg; RLF, 20 deg; L ROT, 48 deg; R ROT, 52 deg; L CFRT 27 deg; R CFRT, 33 deg	Supine left upper cervical rotation clinician mobilization in cervical flexion preposition, 4x5	Produced neck pain, not worse; cervical ROM increased	Education: discontinue if symptoms worsen
	PRO: N/A	Supine left upper cervical thrust mobilization prepositioned in flexion x2	Decreased movement associated neck pain, better; cervical ROM increased	
		Supine left upper cervical rotation MET in supine cervical flexion preposition 3-5 sec, 3x7	Produced neck tightness, not worse; cervical ROM increased	
3-4 (weeks 3-6)	Symptoms: NPRS 3/10	Supine left upper cervical rotation clinician mobilization in cervical flexion preposition, 4x5	Produced neck tightness, not worse; cervical ROM increased	Seated left upper cervical rotation with self-overpressure in flexion, 10-20 reps every 1-2 hours
	Key baselines: EXT, 40 deg; FLEX, 60 deg; LLF/RLF, 20 deg; L ROT, 45 deg; R ROT, 64 deg; R/L CFRT, 37 deg	Supine left upper cervical thrust mobilization prepositioned in flexion x2	Produced neck tightness, not worse; cervical ROM increased	Deep cervical neck flexion endurance training into a pillow, 10 sec x10 reps, once per day
	PRO: NDI 10%	Supine left upper cervical rotation MET in supine cervical flexion preposition 3-5 sec, 3x7	Produced neck tightness, not worse; cervical ROM increased	Education: discontinue if symptoms worsen
		Supine DNF endurance training, biofeedback cuff, at 25 mmHg 10 sec x10		
5-6 (weeks 7-8)	Symptoms: NPRS 1/10	Supine left upper cervical rotation clinician mobilization in cervical flexion preposition, 4x5	Produced neck tightness, not worse; cervical ROM increased	Previous therapeutic exercises and stretching for 2-4 weeks
	Key baselines: EXT, 40 deg; FLEX, 60 deg; LLF/RLF, 20 deg; L ROT, 50 deg; R ROT, 70 deg; R/L CFRT, 37 deg	Supine left upper cervical thrust mobilization prepositioned in flexion x2	Produced neck tightness, not worse; cervical ROM increased	
	PRO: GROC +7/7; NDI 4%	Supine left upper cervical rotation MET in supine cervical flexion preposition 3-5 sec, 3x7	Produced neck tightness, not worse; cervical ROM increased	
		Supine DNF endurance training, biofeedback cuff, 10 sec x10		
		Therapeutic scapulothoracic exercise: upright rows, shoulder press with resistance bands, x30 each		

## Discussion

The cases described above presented to physical therapy with complaints of pain of cervical origin. These presentations followed an initial assessment process utilizing the MDT principles with sagittal and frontal plane repeated end-range movement testing. Symptomatic and mechanical baselines, including the CFRT, were unchanged following repeated sagittal and frontal plane repeated end-range procedures. Favorable outcomes occurred following upper cervical thrust and non-thrust mobilizations followed by rotation DP exercises.

The CFRT has been found to be diagnostic for those patients with cervicogenic headache or temporomandibular joint pain [12,19]. This test is also thought to be influenced by mid- and lower-cervical spine dysfunction, and various cervicothoracic manipulations are indicated in populations with a positive test [8]. These findings may ultimately have implications for the sensitivity, specificity, and construct validity of the test or interventions that are based on a positive test result [20]. Because the utility of using cervical spine arthrokinematics to guide management is debatable, baseline changes in the CFRT were considered in these case to guide cervical rotation-based interventions. These cases demonstrate consideration of cervical rotation DP exercises when DP is not found in testing repeated end-range movements in the sagittal and frontal planes.

This case series describes clinical decision making for patients with neck pain without headache who have a positive CFRT and who do not initially demonstrate favorable changes in baseline measures following repeated movements in the sagittal or frontal planes. The limitations of this study include a small sample size and lack of a control or comparison group. Natural history may have influenced the outcomes reported in this study. Due to interventions by the exclusion of responses to prior movement patterns, in a response-based format, the scope of patients appropriate for these interventions was limited.

## Conclusions

In these cases, the authors found that the CFRT was useful as a clinical baseline prior to and following a repeated movement testing process to determine if a patient demonstrated DP. DP exercises were then prescribed as a part of an HEP, which resulted in favorable outcomes. A larger prospective study would be warranted to examine the prevalence of those meeting the criteria for this proposed subgroup, with comparison of clinical outcomes when utilizing interventions based upon the traditional MDT principle rotation, Orthopedic Manual Physical Therapy (OMPT) principle rotation, or Sustained Natural Apophyseal Glide (SNAG) principle rotation.
